# Rescue of Retinal Degeneration in rd1 Mice by Intravitreally Injected Metformin

**DOI:** 10.3389/fnmol.2019.00102

**Published:** 2019-04-26

**Authors:** Luodan A, Ting Zou, Juncai He, Xia Chen, Dayu Sun, Xiaotang Fan, Haiwei Xu

**Affiliations:** ^1^Key Laboratory of Freshwater Fish Reproduction and Development, Ministry of Education, Laboratory of Molecular Developmental Biology, School of Life Sciences, Southwest University, Chongqing, China; ^2^Southwest Hospital, Southwest Eye Hospital, Third Military Medical University (Army Medical University), Chongqing, China; ^3^Key Lab of Visual Damage and Regeneration & Restoration of Chongqing, Chongqing, China; ^4^Department of Developmental Neuropsychology, School of Psychology, Third Military Medical University (Army Medical University), Chongqing, China

**Keywords:** metformin, retinitis pigmentosa, microglia, crystallin, mice

## Abstract

Retinitis pigmentosa (RP) is a progressive hereditary retinal degenerative disease in which photoreceptor cells undergo degeneration and apoptosis, eventually resulting in irreversible loss of visual function. Currently, no effective treatment exists for this disease. Neuroprotection and inflammation suppression have been reported to delay the development of RP. Metformin is a well-tested drug used to treat type 2 diabetes, and it has been reported to exert beneficial effects in neurodegenerative diseases, such as Parkinson’s disease and Alzheimer’s disease. In the present study, we used immunofluorescence staining, electroretinogram (ERG) recordings and RNA-Seq to explore the effects of metformin on photoreceptor degeneration and its mechanism in rd1 mice. We found that metformin significantly reduced apoptosis in photoreceptors and delayed the degeneration of photoreceptors and rod bipolar cells in rd1 mice, thus markedly improving the visual function of rd1 mice at P14, P18, and P22 when tested with a light/dark transition test and ERG. Microglial activation in the outer nuclear layer (ONL) of the retina of rd1 mice was significantly suppressed by metformin. RNA-Seq showed that metformin markedly downregulated inflammatory genes and upregulated the expression of crystallin proteins, which have been demonstrated to be important neuroprotective molecules in the retina, revealing the therapeutic potential of metformin for RP treatment. αA-crystallin proteins were further confirmed to be involved in the neuroprotective effects of metformin in a Ca^2+^ ionophore-damaged 661W photoreceptor-like cell line. These data suggest that metformin exerts a protective effect in rd1 mice via both immunoregulatory and new neuroprotective mechanisms.

## Introduction

Retinal degeneration (RD), which includes retinitis pigmentosa (RP), features progressive apoptosis and loss of the retinal pigment epithelium (RPE) and photoreceptor cells (Hartong et al., 2006). Thus far, more than 95 mapped and identified genes or genetic loci are involved in autosomal dominant, autosomal recessive and X-linked RP^1^. The disease frequencies of RP varies among isolated populations (Haim, 2002). Currently, no effective treatment exists for this disease. Neurotrophic growth factors were reported to delay the development of RP by rescuing the RPE and photoreceptors from apoptosis (Xu and Peng, 2015). For example, rod-derived cone viability factor (RdCVF) was reported to increase the number of cone cells in P23H RD rats (Ikeda et al., 2014). Moreover, the activation of microglial cells and subsequent inflammation are important pathological changes that are involved in the apoptosis of RPE and photoreceptor cells during the development of RP (Massengill et al., 2018). Therefore, protecting photoreceptors from apoptosis or suppressing inflammation may delay the development of RP.

Metformin is a biguanide hypoglycemic agent that is widely used in the treatment of type 2 diabetes and has been demonstrated to produce anti-inflammatory effects in obesity and cancer (Zhou et al., 2018). Recently, metformin was reported to produce neuroprotective effects against dopaminergic neurodegeneration induced by 3,4-methylenedioxymethamphetamine (MDMA) and against Parkinson’s disease (Porceddu et al., 2016). Long-term metformin treatment has been proven to be helpful in reducing the stroke risk, and this neuroprotective effect results from the reduction in AMP-activated protein kinase (AMPK) by metformin (Deng et al., 2016). Acute preconditioning with metformin also showed a beneficial effect in focal cerebral ischemia through pre-activated AMPK-dependent autophagy (Jiang et al., 2014). Similar mechanisms were involved in the improvement of neurologic function by metformin in cardiac arrest/resuscitation (Zhu et al., 2018)and global cerebral ischemia (Ashabi et al., 2014). As a widely used activator of AMPK, metformin was reported to produce neuroprotection in injury or degeneration of the central nervous system (CNS) mainly through the AMPK-dependent pathway. It is widely known that AMPK is a critical enzyme that maintains cellular energy biogenesis and homeostasis, and it is especially important in neurological function maintenance and neuron survival in the CNS (Xu and Ash, 2016), as the CNS is responsible for more than 50% of total glucose utilization. As evidence, AMPK deficiency was reported to cause severe and rapid neurodegeneration in the retina of Drosophila (Poels et al., 2012). However, it seemed that metformin might produce bi-directional effects on AMPK activation, and reduced pAMPK levels also produced potent neuroprotection (Li et al., 2007; Venna et al., 2012). Further studies are needed to clarify the mechanisms of neuroprotection of metformin.

In addition, reports suggest that there are AMPK-independent pathways in the neuroprotection of metformin. Recent results showed that metformin inhibited the increase in intracellular calcium and reduced cell apoptosis in QUIN-induced neuronal excitotoxicity (Jang and Park, 2018). In methamphetamine-induced neurodegenerative rats, metformin produced neuroprotection by activating the Akt/GSK3 and CREB/BDNF signaling pathways (Keshavarzi et al., 2019). This mechanism is also partially confirmed in oxidatively stressed PC12 cells (Khallaghi et al., 2016). In oxygen-glucose deprivation/reoxygenation-induced neuron injury, metformin protects neurons by downregulating the expression of mitotic arrest deficient 2-like protein 2 (MAD2B) (Meng et al., 2016). Recently, metformin has been reported to improve memory impairment of APP/PS1 Alzheimer’s disease mice through anti-inflammation and clearing amyloid plaque deposition (Ou et al., 2018). The anti-inflammatory effect of metformin was also confirmed in permanent cerebral ischemia (Zhu et al., 2015).

Thus far, the mechanism of metformin’s neuroprotective and anti-inflammatory activity is not fully understood, and the traditional AMPK pathway mechanism is contradictory sometimes. For example, in a cell model and a P23H RP mouse model, metformin improved P23H rhodopsin traffic; however, it also increased photoreceptor cell death due to the instability of metformin-rescued P23H rhodopsin (Athanasiou et al., 2017). Whether metformin produces a neuroprotective effect in other RD mouse models in addition to P23H RP mice is unclear. Rd1 mouse is an animal model of rapid retinal photoreceptor degeneration; the phenotype is mainly caused by mutations in the β subunit of rod cGMP-phosphodiesterase (PDE6β), which results in cGMP accumulation in the retina (Wang et al., 2017), eventually causing early onset severe retinal degeneration (Bowes et al., 1990). Rd1 mice have served as a model for human RP for more than 30 years, and mutations in rd1 mice are homologous to those underlying human pathogenic causes (Li et al., 2016; Xu et al., 2018).

Crystallin proteins, including three major families (α, β, and γ), are highly abundant proteins in the ocular lens. Crystallin proteins were proved to be protective in glaucoma and optic nerve injury, while increased antibodies level against retinal alpha-crystallins usually caused apoptosis of RGCs and optic neuropathy in some glaucoma patients, and the underlying mechanism has not been clarified (Tezel et al., 1998). Our previous results demonstrated that intravitreal injection of α-crystallin promoted axon regeneration in rats through regulation of the RhoA/ROCK (Rho-associated kinase)/cofilin/MCL signaling pathway (Wang et al., 2012). αB-crystallin was reportedly involved in autophagy in the RPE (Zigler et al., 2011). alpha-crystallin were reported to interact with apoptotic, inflammatory and growth factor molecules, while αB-crystallin was shown to regulate insulin, IGF-1 and other growth factors and apoptosis-related proteins (Ghosh et al., 2007).

The crystallin and antiapoptotic genes were reported to be significantly upregulated by intravitreally transplanted adult bone marrow-derived hematopoietic stem cells in the retina of rd1 and rd10 mice. This treatment exerts neuroprotective and neurotrophic effects and delays the progression of RP in rd1 and rd10 mice (Otani et al., 2004). Studies have shown that crystallin proteins can inhibit photoreceptor cell apoptosis *in vivo* and *in vitro* (Hamann et al., 2013; Xu et al., 2015). As both metformin and crystallin proteins show neuroprotective and anti-neuroinflammatory effects and because they share the same modulation pathways in neurotrophic factors and antiapoptotic pathways, it can be hypothesized that crystallin plays a synergistic role with metformin or might be downstream of metformin.

In the present study, we administered intravitreal injections of metformin to rd1 mice and then evaluated their visual function with light/dark transition tests and electroretinogram (ERG) recordings. Morphological changes in the retina, apoptosis in photoreceptors and the activation of microglia were analyzed in metformin-treated (Met) rd1 mice using immunofluorescence and transferase UTP nick end labeling (TUNEL) staining. RNA-Seq was used to identify the transcription factors and pathways associated with the properties of metformin. In addition, the effect of metformin on photoreceptor apoptosis was verified in the 661W photoreceptor-like cell line *in vitro*. Our results reveal a novel strategy for treating RD.

## Materials and Methods

### Animals

Specific pathogen-free (SPF) grade B6.C3-Pde6brd1Hps4le (rd1) and C57BL/6J (C57) mice were supplied by the Experimental Animal Center of the Army Military Medical University and raised in the Experiment Animal Center of Southwest Hospital (the First Affiliated Hospital of the Army Military Medical University). The breeding environment was controlled at 23–25°C, the relative humidity was controlled at 60–70%, the noise was kept at ≤60 dB, and the standard was a 12/12 h day/night cycle, with the light intensity controlled at 150–300 lx. The animals had *ad libitum* access to food and water. All experiments and related measures were performed according to the regulations of the Laboratory Animal Management and Use Committee of Army Military Medical University and the Constitution of the Laboratory Animal Welfare and Ethics Committee of the Army Military Medical University.

### Intravitreal Injection

**FIGURE 1:**
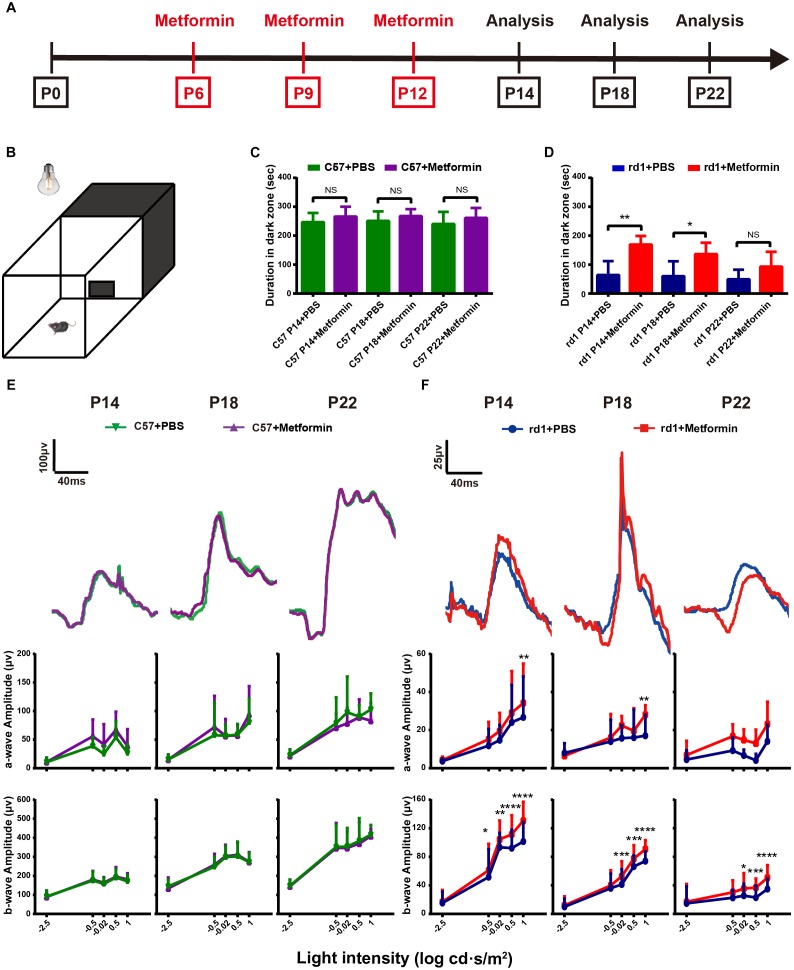
Intravitreal injection of metformin significantly improves the visual function of rd1 mice. Light/dark transition tests and ERG tests were performed at P14, P18, and P22 in rd1 and C57 mice. **(A)** This diagram represents the experimental protocol timeline. **(B)** The light/dark transition test box comprised a dark compartment (one-third of the floor area) and a larger illuminated compartment (two-thirds of the floor area). There was a small opening in the ground plane in the middle of the dividing wall that allowed the mouse to pass freely from box to box. **(C,D)** The time of duration in the dark zone in C57 and rd1 mice treated with PBS or metformin. *n* = 5 mice per group. **(E,F)** ERG waveforms at the 0.5 log cd⋅s/m^2^-light intensity and a comparison of a-and b-wave amplitudes in the control and metformin-treated groups at different periods. **(E)** C57 mice; **(F)** rd1 mice. ^∗^*P* < 0.05, ^∗∗^*P* < 0.01, ^∗∗∗^*P* < 0.001, ^∗∗∗∗^*P* < 0.0001. *n* = 5 mice per group.

Metformin (Sigma-Aldrich, St. Louis, MO, United States) was liquified in phosphate buffer (Phosphate-Buffered Saline, PBS, HyClone, GE Healthcare Life Sciences, Pittsburgh, PA, United States) at a concentration of 20 μg/μl. The day of birth of the mouse was recorded as day 0 after birth (P0). rd1 and C57 mice at P6, P9 and P12 were intraperitoneally injected with 1% pelltobarbitalum natricum (2.5 ml/kg) (Thermo Fisher, Waltham, MA, United States), and oxybuprocaine hydrochloride was then dropped onto the surface of the eyes. A metformin solution (0.5 μl of a 20 μg/μl solution) was then injected intravitreally with a micro-syringe (33G; Hamilton, Bonaduz, Switzerland) into one eye, and the other eye was injected with an equal amount of PBS and used as a control (He et al., 2017). After the operation, the eye was coated with tobramycin dexamethasone eye ointment (Dian Bishu) (Oubaha et al., 2016). Bilaterally treated rd1 mice were tested on P14, P18, and P22 after metformin or PBS administration only in the light/dark transition tests (Figure 1A).

### Immunofluorescence Staining and Terminal Deoxynucleotidyl TUNEL Assays

The mice were sacrificed at the end of the experiments, and the eyeballs were isolated and placed in 4% paraformaldehyde (PFA) for 30 min at room temperature. The cornea and lens were then removed under a microscope (Olympus, Tokyo, Japan) and placed in PFA for 1.5 h. The eyeballs were transferred to 30% sucrose for dehydration and cryopreservation overnight and then placed in Optimal Cutting Temperature (OCT) (Sakura FineTek, Torrance, CA, United States) at -80°C to obtain frozen sections. The sections were cut to be 10 μm thick in the sagittal plane using a cryostat (Thermo Fisher) according to previously described experimental requirements (Chen D. et al., 2016; Chen X. et al., 2016), and serial sections through the pupil-optical axis were collected. Finally, the sections were stored at -20°C. Immunofluorescence staining of the retina was performed as previously described (Li et al., 2016). For slides, we fixed cells with 4% PFA for 20 min at 4°C. In brief, the sections and slides were washed three times with PBS and then incubated with 0.3% Triton X-100 and 3% bovine serum albumin (BSA) for 30 min at 37°C. The sections and slides were then immersed in primary antibodies diluted in 1% BSA + 0.1% Triton and incubated overnight at 4°C. The sections and slides were washed three times with PBS and then incubated with fluorophore-conjugated secondary antibodies diluted in PBS for 1 h at 37°C (Table 1). For TUNEL staining, buffers 1 and 2 (*In Situ* Cell Death Detection Kit, Roche, Basel, Switzerland) were mixed at a ratio of 1:9 according to the manufacturer’s instructions and added to the sections and slides, which were then incubated for 2 h at 37°C. The sections and slides were washed four times with PBS (10 min/time), and the nuclei were counterstained with a dimercaptophenyl hydrazine solution (4′,6-diamidino-2-phenylindole, DAPI; Sigma). The sections and slides were then visualized, and images were acquired and quantified using a confocal microscope (Zeiss LSM 800 confocal microscope, ZEN Microsystems; ZEISS, Germany).

**Table 1:** List of antibodies.

Name	Application	Host	Supplier
**Primary** **antibodies**			
GNAT1	IF(1:500)/WB(1:2000)	Rabbit	Santa Cruz
Rhodopsin	IF(1:500)	Mouse	Abeam
PKCa	IF(1:500)/WB(1:2000)	Rabbit	Abeam
Ibal	IF(1:500)/WB(1:2000)	Rabbit	Abeam
aA-crystallin	IF(1:500)/WB(1:2000)	Mouse	Santa Cruz
Anti-cone arrestin	IF(1:500)	Rabbit	Millipore
P-actin	WB(1:2000)	Mouse	Sigma
**Secondary antibodies**			
Anti-mouse IgG Alexa Fluor^®^568	IF(1:500)	Goat	Thermo Fisher Scientific
Anti-rabbit IgG Alexa Fluor^®^568	IF(1:500)	Goat	Thermo Fisher Scientific
Anti-mouse IgG Alexa Fluor^®^488	IF(1:500)	Goat	Thermo Fisher Scientific
Anti-rabbit IgG Alexa Fluor^®^488	IF(1:500)	Goat	Thermo Fisher Scientific
Anti-rabbit IgG Alexa Fluor^®^647	IF(1:500)	Goat	Thermo Fisher Scientific
Anti-mouse IgG HRP	WB (1:2000)	Goat	Beyotime
Anti-rabbit IgG HRP	WB (1:2000)	Goat	Beyotime


### Fluorescence Intensity Analysis

As described previously, the mean fluorescence pixel intensity of the markers to be detected in the images were detected by ImageJ software after the background was subtracted (National Institutes of Health, Bethesda, MD, United States) (Akopian et al., 2014). Each image was detected at least three times, and the average of the obtained data was taken as a mean fluorescence pixels intensity. Each dataset came from at least five samples.

### Electroretinogram (ERG)

The visual electrophysiological tests were performed as described previously (Dai et al., 2017a,b). The recordings were made between 7:30 and 11:30 PM. Before the test, the mice (*n* = 5 for each time point) were adapted to darkness for nearly 12 h under dim red light, and 1% pelltobarbitalum natricum (2.5 ml/kg) was intraperitoneally injected for anesthesia. After anesthetization, the mice were placed on the electrophysiological instrument with a heating pad maintained at 37°C. The pupils were dilated with one drop of tropicamide eye drops (Santen Pharmaceutical, Osaka, Japan). The ERG responses of both eyes were recorded with light guide electrodes that were placed in contact with the cornea; reference electrodes were placed subcutaneously in the forehead area, and ground electrodes were inserted subcutaneously into the tails of the mice. An appropriate eye ERG device (Diagnosys LLC, Lowell, MA, United States) was used to measure scotopic ERG recordings at light intensities of -2.5, -0.5, -0.02, 0.5, and 1 log candela⋅s/meter^2^ (log cd⋅s/m^2^). Responses to 3 light flashes were averaged at the lower light intensities (-2.5 log cd⋅s/m^2^), whereas only 1 light flash was applied for the higher light intensities (-0.5, -0.02, 0.5, and 1 log cd⋅s/m^2^). The recovery periods between different light intensities were arranged as follows: 30 s, 1 min, 2 min, 3 min, and 4 min. The amplitudes and implicit times of the a-waves and b-waves were recorded using Diagnosys Electrophysiology System software V6. The data were analyzed with GraphPad Prism 6(McCulloch et al., 2015).

### Light/Dark Transition Test

The light/dark transition tests were performed as previously described (Chen D. et al., 2016; Chen X. et al., 2016). The volume of the dark and light box was 45 cm × 30 cm × 40 cm, and there was a 10 cm × 10 cm gap between the light room (30 cm × 30 cm × 40 cm) and the dark room (15 cm × 30 cm × 40 cm). The mice were dark-adapted for at least 12 h. The channel between the light chamber and the dark chamber was blocked before the experiment began. The mice were first placed in a dark room for 2 min. The space between the light and dark rooms was then opened, and the mice were allowed to move freely between the light and dark rooms for 5 min. A tungsten light bulb with a light intensity of 300 lux was hung in the area directly above the light room. The activity of the mice in the light room was recorded with a camera and statistically analyzed.

### RNA-Seq and Bioinformatic Analysis

The mice were treated as described above, and retinal tissue samples were taken at P14. For convenience, we labeled the metformin treatment group as “Met” and the PBS treatment group as “control,” and in each group three biological replicates were used for the sample analysis.

RNA from the two groups was extracted using TRIzol^TM^ (Invitrogen, Carlsbad, CA, United States). RNA was used for mass detection using an Agilent 2100 Bioanalyzer (Agilent RNA 6000 Nano Kit). Library construction and sequencing were performed on a BGISEQ-500 by Genomic Institution. Gene expression levels were shown as the FPKM value. We detected differentially expressed genes (DEGs) with DEG-seq as requested and defined fold changes ≥ 2 and adjusted *P*-values ≤ 0.001 as indicative of DEGs.

The genes with significant differences between the experimental group and the comparison group were obtained by high-throughput gene sequencing analysis, and then the Kyoto Encyclopedia of Genes and Genomes (KEGG) pathway was enriched to obtain a significantly enriched KEGG pathway in the differential gene. According to the interaction network between the pathways in the KEGG database^2^, the interactions of the obtained differential genes significantly enriched in the KEGG pathway were extracted from the database. Then, these interaction relationship files were imported into Cytoscape 3.5.0 software and drawn into a KEGG path interaction network diagram. In the article, the complete picture was placed in the Supplementary Material, and the key part of the screenshot was enlarged and placed in the text for a clearer explanation of possible core paths.

A pathway analysis was performed using the KEGG database^3^ based on DEGs. An annotation analysis was performed through Gene Ontology (GO)^4^ for the screened DEGs. A “pathway-act-network” analysis (KEGG database) and a protein–protein interaction analysis of the DEGs (String database) were constructed with Cytoscape 3.0 (Sun et al., 2017).

### Real-Time Quantitative Polymerase Chain Reaction (RT-qPCR)

The RT-qPCR tests were performed as described previously (He et al., 2017). In brief, total RNA was extracted from each retina or 1 × 10^6^ cells with 1 mL of TRIzol^TM^ (Sigma-Aldrich, St. Louis, MO, United States), 200 μL of chloroform, 500 μL of isopropanol and 1 mL of 75% ethyl alcohol. The concentration and purity of the RNA were detected using a spectrophotometric instrument (Thermo Fisher). Following the manufacturer’s instructions, reverse transcription was performed using a Prime Script RT Reagent Kit (Takara, Tokyo, Japan) and qPCR was carried out with SYBR Green qPCR Mix (Takara Bio Inc., Japan) through a CFX96 Real-Time PCR System (Bio-Rad, Hercules, CA, United States). The primers were produced by Sangon Biotech (Shanghai, China), and they are shown in Table 2. The PCR conditions were as follows: 30 s at 95°C, 41 cycles of 5 s at 95°C and 30 s at 60°C followed by plate reading and then 10 s at 95°C followed by a melting curve analysis (65–95°C in increments of 0.5°C per 5 s).

**Table 2:** List of Primers.

Name	Forward primer	Reverse primer
Cryaa	*CAGCATCCTTGGTTCAAGCG*	GGCGGTAGTAGGGGCTGAT
Crybal	*TGAGCGTCTCATGTCCTTCC*	CCACTGGCGTCCAATAAAGTTC
Cryba2	*AGAGAAGGGAGACTATCCTTGC*	CGGAGCTGACTTTCAGGGAAC
Cryba4	*GTGTCCTGGAACTTGGTTTTGA*	CGAAGATGGTCAGCCTTGAGT
Crybbl	*TGCCAAAGTAGGGGACCTG*	CGGAAGGCAGATTGCTCAAAG
Crybb2	*CAAGGGCGAGCAGTTTGTG*	CGAAGATGGTCAGCCTTGAGT
Crybb3	*CGGAGTGTCCCAACCTCAC*	AACAAACTGCTCCCCACGAAA
Cryga	*TCCATTCCATACACCAGCTCT*	GCAGGAACAGTCGTCCATGAG
Crygb	*ACAAATGTCAGAGATCA CAGACG*	GACTCGCCTAAAAGAGCCAAC
Crygc	*AATGCGGCTGTATGAGAAAGAA*	GGAAGCGCCGGTACTCTTG
Crygd	*CGGCTCTCACAGGA TCAGACT*	GGTAGTTGGTCATGTCGT AGAGG
Cryge	*ACCAGCAGTGGA TGGGTTTC*	GTGGGAGCAGTCGTCTGTGAT
Crygf	*TCACAGGATCAGGA TCTACGAG*	GGACCCAGTAGCCCTCCAT
Crygn	*GCCAGTGCCTAGAGTTCGTG*	AAGCAGCGGTAGTCTCCTCTC
Crygs	*CAGACTTCCGCTCGTACCTAA*	TCGCCCTGGGGTAAGATGT
IL10	*CAGTACAGCCGGGAAGAC AATAA*	CCGCAGCTCTAGGAGCATGT
IL4	*TCCTGCTCTTCTTTCTCG*	TTCTCCTGTGACCTCGTT
Birc3	*CTTTGAGCACAAGTCCCT ACCAC*	AGCATCATCCTTACGTTCCCAGT
Birc5	*GAGGCTGGCTTCATCCACTG*	ATGCTCCTCTATCGGGTTGTC
Bakl	*TCCTGACAGACGGACGGA CAGAG*	GGCAGACAAGGAGTGAA GGTGGG
Tgfbl	*TGGAGCAACATGTGGAACTC*	GTCAGCAGCCGGTTACCA


### Western Blot Analysis

A Western blotting analysis was performed following our previously described methods (Chen D. et al., 2016; Chen X. et al., 2016). Retinas were isolated and lysed in ice-cold tissue lysis buffer (1% PSMF + 99% RIPA). For cells, the cell pellet was added directly to the lysis buffer, and the mixture was then sonicated under an ice bath to allow the cells to fully lyse. After centrifugation at 2,000 × *g* at 4°C for 30 min, the protein in supernatants was collected, and the concentration of protein was determined using a bicinchoninic acid assay (BCA) (Beyotime, China). As previously reported (Chen D. et al., 2016), a 30 μg protein sample from each group was loaded and separated using a 15% sodium dodecyl sulfate (SDS) polyacrylamide gel. Then, the proteins were transferred to polyvinylidene fluoride (PVDF) membranes (Bio-Rad). After blocking with 5% non-fat milk in Tris-buffered saline/Tween (TBST), the membranes were incubated with primary antibodies at 4°C overnight. The next day, the membranes were washed with TBST three times and then incubated with peroxidase-conjugated immunoglobulin G (1:2000; Beyotime, China) as a secondary antibody for 2 h at room temperature. Finally, the protein bands were imaged using the enhanced chemiluminescence method (Amersham, Piscataway, NJ, United States) following the manufacturer’s instructions and scanned with an Odyssey infrared imager system. Protein staining was quantified and analyzed by ImageJ software using β-actin as control.

### Cell Culture and Processing

The 661W cells were provided by Dr. Luo of the State Key Laboratory of Ophthalmology, Zhongshan Ophthalmic Center, Sun Yat-sen University, Guangzhou, Guangdong, China (Ma et al., 2015). The cell cultures were prepared as described previously (Wang et al., 2012). In brief, the cells were seeded in 60-mm dishes and cultured in a 37°C incubator containing 5% CO_2_. The medium was Dulbecco’s Modified Eagle’s Medium (DMEM; HyClone) containing 10% fetal bovine serum (FBS, Thermo Fisher) and 1% penicillin-streptomycin (Thermo Fisher). In all cell experiments, 2 × 10^5^ cells were inoculated into one well of a six-well plate. After adherent growth was allowed for 16 h, the wells were washed three times with PBS, and the remaining cells were divided into 4 groups: (1) control, (2) Met, (3) Ca^2+^ ionophore (0.5 μM) and (4) Ca^2+^ ionophore + Met (1 mM). The cells were then allowed to further grow in a serum-free medium. An equal volume of DMSO was added to the control group, the Met group and the Ca^2+^ ionophore group. After culturing for 24 h, the cells were collected for testing.

### Cell Viability Assay

We performed a Cell Counting Kit-8 (CCK-8; Dojindo Laboratory, Japan) assay according to the manufacturer’s instructions to measure cell viability. A total of 1 × 10^4^ cells were added to each well of a 96-well plate and processed using a cell viability assay as previously described. In brief, the medium was aspirated and the mixture of the CCK8 reaction solution and the new serum-free medium at a 1:10 ratio was added to the 96-well plate and cultured at 37°C for 1 h in the dark. The absorbance was measured at 450 nm with a microplate reader (Varioskan Flash, Thermo Fisher).

### Statistical Analysis

The results of the light/dark transition test and ERG analysis are presented as the mean ± SD, and the other results are presented as the mean ± SEM. Paired *T*-tests and independent sample *t*-test followed by Fisher’s protected least-significant difference *post hoc* tests were used for RT-qPCR, histological analysis, ERG analysis, Western blotting, and light/dark transition test analyses. For comparisons among groups in cell experiments, one-way ANOVA followed by Fisher’s protected least-significant difference *post hoc* test was used. For all comparisons in this study, the significance level was set at *P* < 0.05. The statistical figure was drawn using Prism 6.01.

## Results

### Metformin Delays Visual Impairment in rd1 Mice

The light/dark transition test and ERG tests were performed to detect the effects of metformin on visual function in rd1 mice, with C57 wild-type mice as the control (Figure 1A). The literature reports that natural spectra may have an effect on mouse retinal rod cells, cones, and ipRGCs (Pearson et al., 2012). The main photoreceptors type in the mouse retina is rod cells. In the rd1 model, the rod-specific gene mutation led to rod death initially, but cone degeneration occurred subsequently. Although cone death was obviously observed from P35, some underlying alterations of the cones, such as changes in the glutamate cysteine ligase content, occurred earlier, which may influence the light-triggered aversion response (Punzo and Cepko, 2007; Sanchez-Vallejo et al., 2016). Interestingly, our results showed the decrease in cone-mediated function at a relatively early stage, and metformin was found to alleviate the loss in function. To interpret the results more precisely, we analyzed the duration in the dark zone instead of previous time in light (Figure 1C,D). At P14, P18, and P22, the mice were placed in the apparatus as shown in Figure 1B for 5 min, and the time in the dark zone was recorded. At P14, P18, and P22, there were no differences in time in the dark zone between the Met and PBS-treated C57 mice, indicating that metformin did not affect normal visual function (Figure 1C). Moreover, the rd1 mice treated with PBS spent less time in the dark zone, whereas metformin treatment markedly increased the time in the dark zone. These results demonstrate that metformin treatment improved the visual function of rd1 mice at P14 (*P* < 0.01) and P18 (*P* < 0.05). However, at P22, the difference between the metformin- and PBS-treated groups was not significant (*P* = 0.15), with both groups of rd1 mice having very weak light perception (Figure 1D) (*n* = 5). This finding suggests that metformin improved the visual function of rd1 mice and did not influence normal mice.

The visual function of the retina can be further evaluated using ERG as the activity of the retina as a whole after light stimulation (McCulloch et al., 2015). Consistent with the results of the light/dark transition, there was no significant difference in the amplitudes or the latencies of a- and b-wave between Met- and PBS-treated C57 mice at P14, P18 and P22 (Figure 1E and Supplementary Figure S1A). In rd1 mice, a-wave amplitudes at 1 log cd⋅s/m^2^ light intensity were significantly better in the Met group than in the PBS-treated group at P14 and P18. Notably, in rd1 mice, the b-waves were higher in the Met group than in the PBS-treated group, and these increases were significant at -0.5, -0.02, 0.5, and 1 log cd⋅s/m^2^ at P14; at -0.02, 0.5, and 1 log cd⋅s/m^2^ at P18 and P22 (*n* = 5) (Figure 1F). However, there was almost no significant difference in the latency of a- and b-wave between the PBS-treated and Met rd1 mice (Supplementary Figure S1B). These data indicate that injecting metformin protected visual function in rd1 mice.

### Metformin Rescues Photoreceptors From Apoptosis in rd1 Mice

**FIGURE 2:**
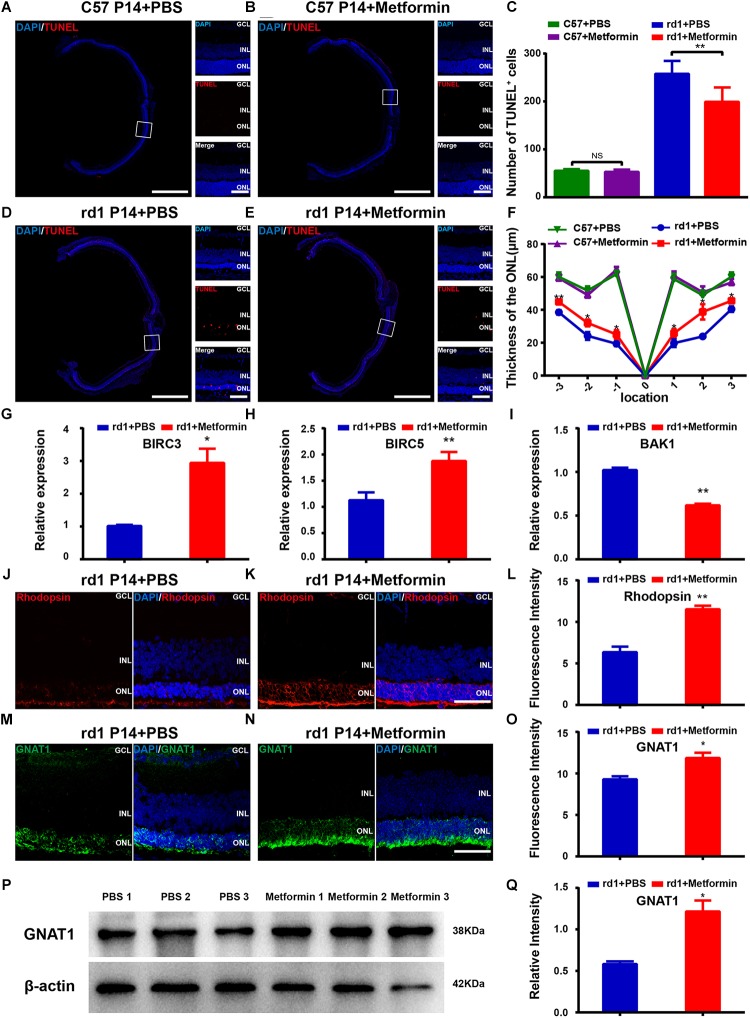
Metformin rescues photoreceptors from apoptosis in rd1 mice at P14. **(A,B,D,E)** Whole retinal montage and partial enlargement of TUNEL staining in the following differently treated groups of mice: **(A)** C57 mice treated with PBS; **(B)** C57 mice treated with metformin; **(D)** rd1 mice treated with PBS; **(E)** rd1 mice treated with metformin. A magnified view from a C57 mouse was obtained approximately 450 μm from the optic papilla, whereas the view obtained from the rd1 mouse was located approximately 400 μm from the optic papilla. **(C)** Comparison of the numbers of TUNEL-positive cells in the whole retina in C57 and rd1 mice in two treatment groups. *n* = 7 mice per group. **(F)** Comparison of ONL thickness at different positions of the retina in C57 and rd1 mice. *n* = 5 mice per group. **(G–I)** Real-time PCR analysis of the expression of BIRC3, BIRC5, and BAK1. *n* = 3 mice per group. Representative images of immunofluorescence for specific markers of photoreceptor cells (green/red) and DAPI (blue) in the mouse retina showing that the retina expresses Rhodopsin (**J,K** in red), GNAT1 (**M,N** in green): **(G)** Rd1 mice treated with PBS at P14; **(H)** rd1 mice treated with metformin at P14; **(J)** rd1 mice treated with PBS at P14; **(K)** rd1 mice treated with metformin at P14. **(L,O)** Quantitative analysis of the intensities of Rhodopsin **(I)** and GNAT1 **(L)** expression in the two groups shown in **(G,H,J,K)**. *n* = 5 mice per group. **(P)** Representative Western blot bands of GNAT1 versus β-actin. **(Q)** Comparison of protein grayscale semiquantitative analysis between the control group and the Met group. GCL, retinal ganglion cell layer; INL, inner nuclear layer; ONL, outer nuclear layer. Data are presented as the mean ± SEM. NS, no significant difference, ^∗^*P* < 0.05, ^∗∗^*P* < 0.01. Scale bars: whole retinal map, 500 μm; enlarged view, 50 μm; **(G,H,J,K)** 50 μm.

We further studied the effect of metformin on photoreceptors in the retina of rd1 mice. As photoreceptor apoptosis starts at P8 and reaches a peak at P14 in the retina of rd1 mice (Li et al., 2016), we selected a time close to P8 to intravitreally inject metformin and analyzed the effect at P14. TUNEL staining was used to label apoptotic cells in the retina. The results revealed a small number of apoptotic cells in the retina of C57 mice. In rd1 mice at P14, most of the apoptotic cells in the retina were localized in the outer nuclear layer (ONL) (Figure 2A,B,D,E). There were very few apoptotic cells in the retina of P14 C57 mice, and metformin pretreatment did not alter this number (*P* > 0.05). However, at P14, the number of apoptotic cells in the ONL was significantly higher in rd1 mice than in age-matched C57 mice, and treatment with metformin significantly reduced the increase in apoptotic cells in the ONL of rd1 mice (*P* < 0.05, *n* = 7) (Figure 2C). Metformin treatment had no obvious impact on the thickness of the ONL at any site in C57 mice (*P* > 0.05). In P14 mice, the thickness of the ONL was significantly smaller in PBS-treated rd1 mice than in age-matched C57 mice, and treatment with metformin significantly inhibited the thinning of the ONL in rd1 mice (*P* < 0.01, *n* = 5) (Figure 2F). To further examine the effect of metformin on retinal apoptosis in rd1 mice, we used RT-qPCR to detect the expression of some important -related genes in mouse retina after drug treatment. We found that the expression levels of the anti-apoptotic genes baculoviral IAP repeat-containing 3 (BIRC3), baculoviral IAP repeat-containing 5 (BIRC5) were significantly upregulated and the pro-apoptotic gene BCL2-antagonist/killer 1 (BAK1) was significantly downregulated after metformin treatment compared with the PBS-treated group (Figure 2G–I). These results show that metformin rescued apoptotic photoreceptors and delayed the degeneration of the retina in rd1 mice.

Rhodopsin and GNAT1, markers of photoreceptors, were used to observe the effect of metformin treatment on photoreceptor cells (Figure 2J,K,M,N). As the retina undergoes degeneration in rd1 mice, apoptosis gradually increases in the photoreceptor cells of the retina. In these experiments, we compared the fluorescence intensities of rhodopsin and GNAT1 labeling in the retina of PBS-treated and metformin-treated rd1 mice at P14. The results showed that the fluorescence intensities of rhodopsin (*P* < 0.05) and GNAT1 (*P* < 0.05) were significantly higher in the Met group than in the PBS-treated group (*n* = 5) (Figure 2L,O). In addition, the Western blotting results confirmed the effect of metformin on GNAT1 levels in the retina of rd1 mice (*P* < 0.05, *n* = 3) (Figure 2P,Q). These data suggest that metformin protects photoreceptor cells from apoptosis in rd1 mice.

The change in bipolar cells, another type of retinal neuron that expresses PKCα, was also studied after metformin treatment. The results show that the fluorescence intensity of PKCα was not significantly different between the metformin-injected group and the PBS-injected group (*P*> 0.05, *n* = 5) (Supplementary Figures S2A–C). The Western blot results confirmed this phenomenon (*P* > 0.05, *n* = 3) (Supplementary Figures S2D,E). These results indicate that metformin has no significant effect on bipolar cells in the retina of rd1 mice at P14.

### Metformin Inhibits the Activation of Microglia and Reduces Inflammation in rd1 Mice

**FIGURE 3:**
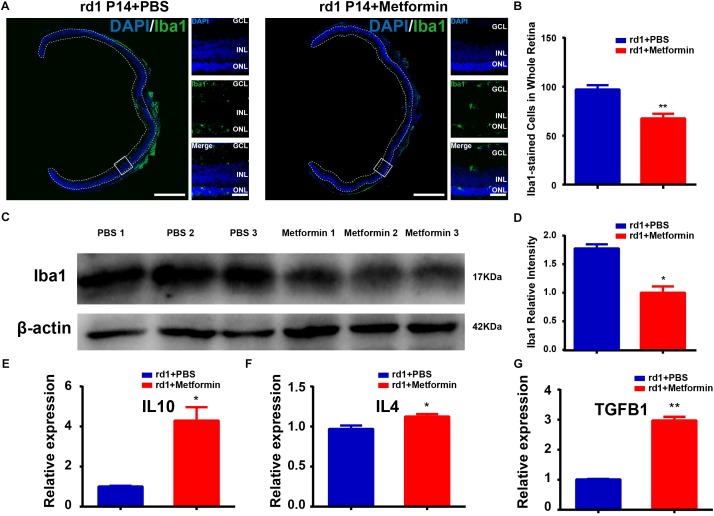
Metformin inhibits the activation of microglia and inflammation in rd1 mice at P14. **(A)** Whole retinal montage and partial enlargement of an image showing Iba1 staining in the control and Met groups. **(B)** Comparison of the number of Iba1-positive cells in the whole retina. *n* = 5 mice per group. **(C)** Representative Western blot bands of Iba1 versus β-actin. **(D)** Comparison of protein grayscale semiquantitative analysis between the control group and the Met group. *n* = 3 mice per group. **(E–G)** Real-time PCR analysis of the expression of IL10, IL4, and TGFB1. *n* = 3 mice per group. ^∗^*P* < 0.05, ^∗∗^P < 0.01. Scale bar: whole retinal map, 500 μm; enlarged view, 50 μm.

Microglial activation is thought to be a hallmark of neuroinflammation. During the retinal degeneration of rd1 mice, microglia are gradually activated, becoming amberoid-activated shaped cells with round bodies and few dendrites (Li et al., 2016; He et al., 2017). At P14, most of the photoreceptor cells in the ONL are undergoing apoptosis, and the number of microglia reaches its peak, with activated microglia migrating to the ONL (Zhou et al., 2017). Iba1-positive microglia were previously shown to be distributed throughout the retina and exhibit an amoeba-like activation state in the retina of rd1 mice. Metformin treatment markedly suppressed microglial activation (Figure 3A). The number of Iba1-positive microglia was significantly lower throughout the retina in the Met group than in the PBS treatment group (*P* < 0.01, *n* = 5) (Figure 3B). Western blotting for Iba1 confirmed lower levels in the retina of the Met rd1 group (*P* < 0.05, *n* = 3) (Figure 3C,D). To elucidate the effect of metformin on retinal inflammation in rd1 mice, we used RT-qPCR to study the expression of important inflammatory genes in the retina after drug action. We found that the expression levels of the anti-inflammatory genes interleukin 10 (IL10), interleukin 4 (IL4) and transforming growth factor beta 1 (TGFB1) in the retina treated with metformin significantly decreased compared with the PBS-treated group (Figure 3E–G). These data indicate that metformin inhibited the activation of microglia and reduced inflammation in the retina of rd1 mice.

### Changes in the Gene Expression Profile of rd1 Mice After Metformin Treatment

To increase our understanding of the mechanisms by which metformin delays visual function impairment in rd1 mice, exploring the changes that occur in the gene expression profile of the rd1 mouse retina is necessary. Therefore, mRNA sequencing was performed in the retina of rd1 mice. A total of more than 6.8 billion clean reads were generated from all six cDNA libraries using the BGISEQ-500 platform. The gene expression profile of the metformin-treated rd1 mice retina was compared with that of the PBS-treated rd1 mouse retina. For convenience, we refer to the metformin group as “Met” and the PBS treatment group as “control.” Fold changes ≥ 2 and adjusted *P*-values ≤ 0.001 for gene expression indicated significantly differential gene expression.

**FIGURE 4:**
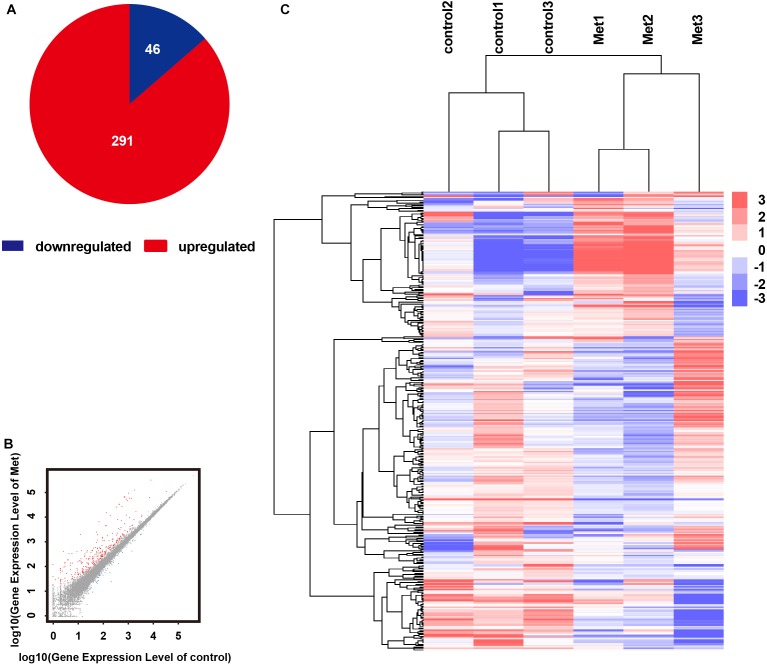
RNA sequencing results showing that metformin affected gene expression in the rd1 mice retina at P14. **(A)** Pie chart of all differentially expressed genes. red: gene upregulated by metformin; blue: gene downregulated by metformin. **(B)** Scatterplots showing all expressed genes as the control group expression level vs. the metformin-treated group expression level. red: gene upregulated by metformin; blue: gene downregulated by metformin; gray: gene not regulated by metformin. **(C)** Hierarchical clustering of differentially expressed genes in the control and metformin treatment groups. Red: high relative expression; blue: low relative expression. *P*-values and *Q*-values (MA-plot) represent the significance of the differences (fold change ≥ 2 and adjusted *P*-value ≤ 0.001).

**FIGURE 5:**
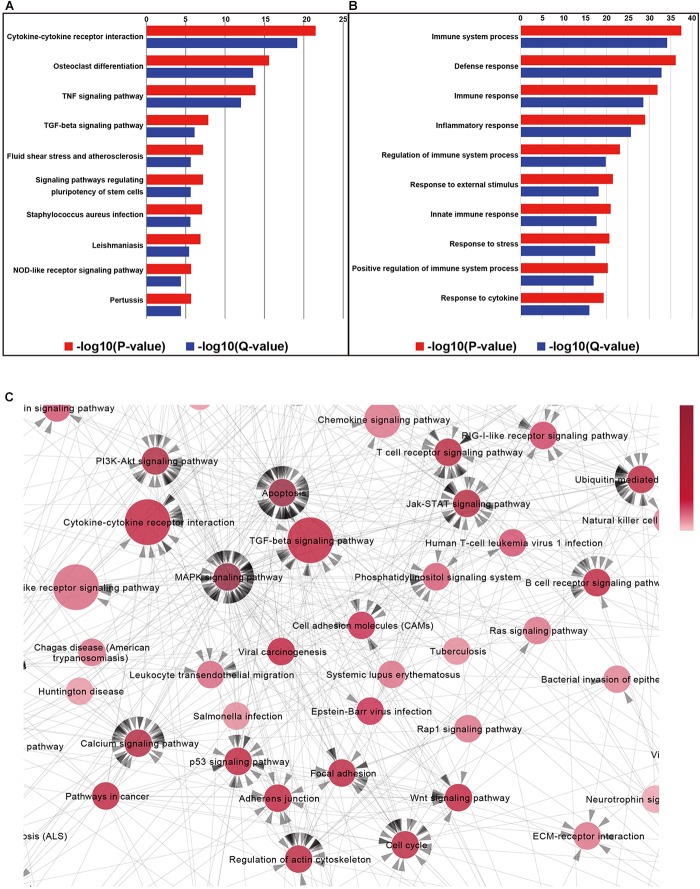
Column charts of the Gene Ontology (GO) and pathway enrichment analyses of differentially expressed genes in the retina of metformin-treated rd1 mice at P14. **(A)** The 10 most significant biological processes in the GO enrichment analyses. *P*-values and *Q*-values represent the level of significance of enrichment. **(B)** The 10 most significant pathways in the Kyoto Encyclopedia of Genes and Genomes (KEGG) pathway enrichment analyses. **(C)** Partial enlargement of the pathway-act-network analysis of the retina in metformin-treated and PBS-treated rd1 mice at P14. A pathway-act-network was constructed according to the interactions within pathways identified in the KEGG database. Each node (red circle) represents a signaling pathway. Arrows represent interactive relationships between two signaling pathways. We used the size of the circle to represent the significance of the difference between the groups in the KEGG pathway. The circular red shades represent the clustering coefficients. A darker color indicates more strongly associated pathways. The direction of the arrow represents the upstream and downstream relationship of the path.

The results of mRNA sequencing showed that a total of 337 significantly differentially expressed genes were identified. Among them, 291 were expressed at significantly higher levels and 46 were expressed at significantly lower levels in the Met group than in the control group (Figure 4A,B). The expression levels of the 337 DEGs identified in the control and Met groups were plotted as a heat map (Figure 4C), and KEGG pathway enrichment and GO analyses were performed with EDG. We chose the 10 most significant KEGG and GO pathways, as shown in Figure 5A,B. Immunity and inflammation had relatively important positions in the results of the GO bioanalysis.

To further explore the core pathways underlying the functions of metformin, we constructed a pathway interaction network map based on the relationships among pathways in the KEGG database (Supplementary Figure S3). The core part of the entire network interaction diagram is shown separately (Figure 5C). We used the size of the circle to represent the significance of the difference between the groups in the KEGG pathway. The circular red shades represent the clustering coefficients. A darker color indicates more strongly associated pathways. The direction of the arrow represents the upstream and downstream relationship of the path. After considering the significance of the identified pathway differences and the number of related pathways, we found that the following five KEGG pathways were associated with the effects of metformin on rd1 mice: cytokine-cytokine receptor interactions, the TGF-β signaling pathway, the MAPK signaling pathway, the apoptosis pathway and the PI3K-Akt signaling pathway. It is widely accepted that cytokine-cytokine receptor interactions are associated with the inhibition of inflammation (Veytia-Bucheli et al., 2018), that the TGF-β signaling pathway is related to neuroprotection (Rodriguez-Martinez and Velasco, 2012) and that the activation of the MAPK signaling pathway and the PI3K-Akt signaling pathway can play a neuroprotective role (Zhu et al., 2016). Therefore, the results of KEGG pathway network mapping suggest that neuroprotective and anti-inflammatory effects may be important mechanisms underlying the ability of metformin to delay RP, consistent with our previous results.

**FIGURE 6:**
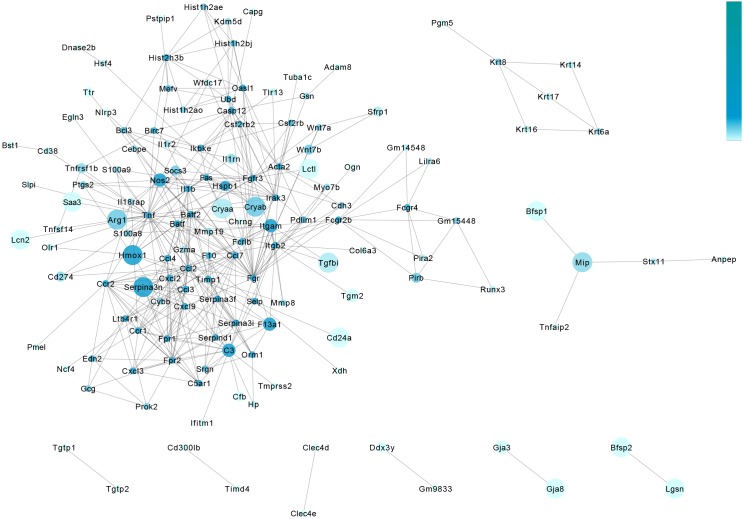
Protein–protein interaction analysis of the retina in metformin-treated and PBS-treated rd1 mice at P14. The gene-act-network was constructed based on the interactions among genes identified in the STRING database. Each node (blue circle) represents a gene. Lines represent interactive relationships between two signaling pathways. We represent the significance of differences in genes in terms of the size of the circle, and the clustering coefficient is represented by the shade of the circle in blue. A darker color indicates more associated genes.

We also constructed a protein–protein interaction analysis of DEGs to further explore the core genes that led to the observed changes in the experimental and control groups. We represent the significance of differences in genes in terms of the size of the circle, and the clustering coefficient is represented by the shade of the circle in blue. A darker color indicates more associated genes (Figure 6).

**FIGURE 7:**
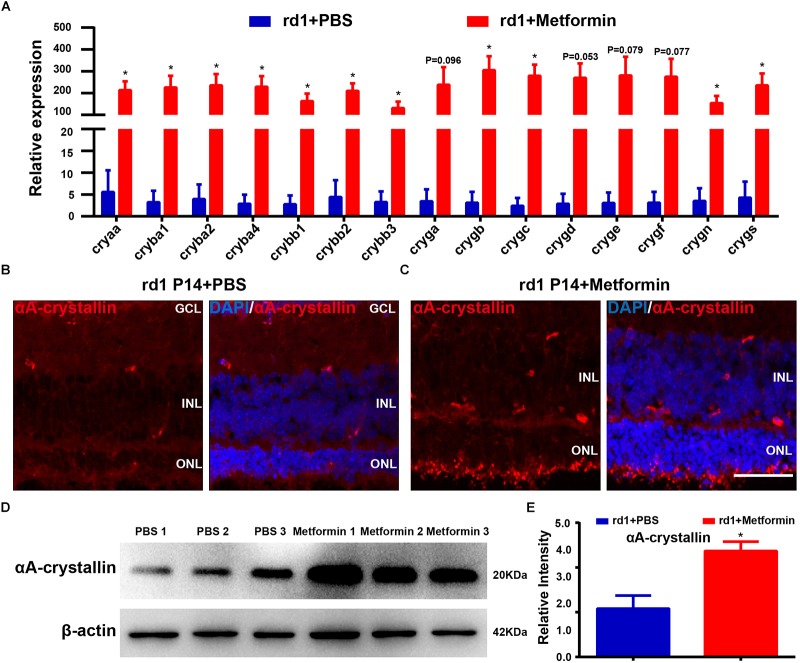
Quantitative RT-PCR validation of core genes identified in the RNA-Seq analysis. **(A)** Real-time PCR analysis of the expression of crystallin. Blue: PBS-treated group. Red: metformin-treated group. Representative images of immunofluorescence for specific markers, including αA-crystallin cells (red) and DAPI (blue), in the mouse retina. **(B)** Rd1 mice treated with PBS at P14; **(C)** rd1 mice treated with metformin at P14. **(D)** Representative Western blot bands for αA-crystallin versus β-actin. **(E)** Comparison of protein grayscale semiquantitative analyses between the control group and the Met group. *n* = 3 samples per group. GCL, retinal ganglion cell layer; INL, inner nuclear layer; ONL, outer nuclear layer. Data are presented as the mean ± SEM. NS, no significant difference. ^∗^*p* < 0.05, ^∗∗^*P* < 0.01. Scale bar: 50 μm.

To validate our mRNA sequencing results, we performed RT-PCR on these core genes. The results show that the expression levels of crystallin were significantly higher in the retina of P14 rd1 mice after metformin treatment than those in the control group (Figure 7A). The genes of crystallin proteins may be the core genes involved in the potential mechanism by which metformin delays RP in rd1 mice. The crystallin introduced in the background can exert anti-inflammatory and antiapoptotic effects in various disease models, and metformin treatment results in the upregulation of anti-inflammatory (IL10, IL4, and TGFB1) and anti-apoptotic genes (BIRC3 and BIRC5) and the downregulation of pro-apoptotic (BAK1), as previously demonstrated. Therefore, we speculated that metformin may exert anti-inflammatory and antiapoptotic effects by upregulating the expression of crystallin proteins, thereby protecting the visual function of rd1 mice.

We next sought to verify our previous results showing that metformin treatment upregulates the expression of crystallin and delays the progression of RP in rd1 mice. We selected a subtype of αA-crystallin, the most prominently differentially expressed crystallin among the DEGs, to analyze the expression and distribution of αA-crystallin using immunofluorescence and Western blot analyses in the retina of P14 rd1 mice. The immunofluorescence results show that αA-crystallin proteins were expressed throughout almost the whole retina (Figure 7B,C). The Western blotting results show that metformin treatment significantly increased the expression of αA-crystallin in the retina of rd1 mice (*P* < 0.05, *n* = 3) (Figure 7D,E), consistent with the results of our previous experiments. Therefore, metformin treatment may delay RP by increasing the expression of crystallin.

### Metformin Protects 661W Cells *in vitro*

**FIGURE 8:**
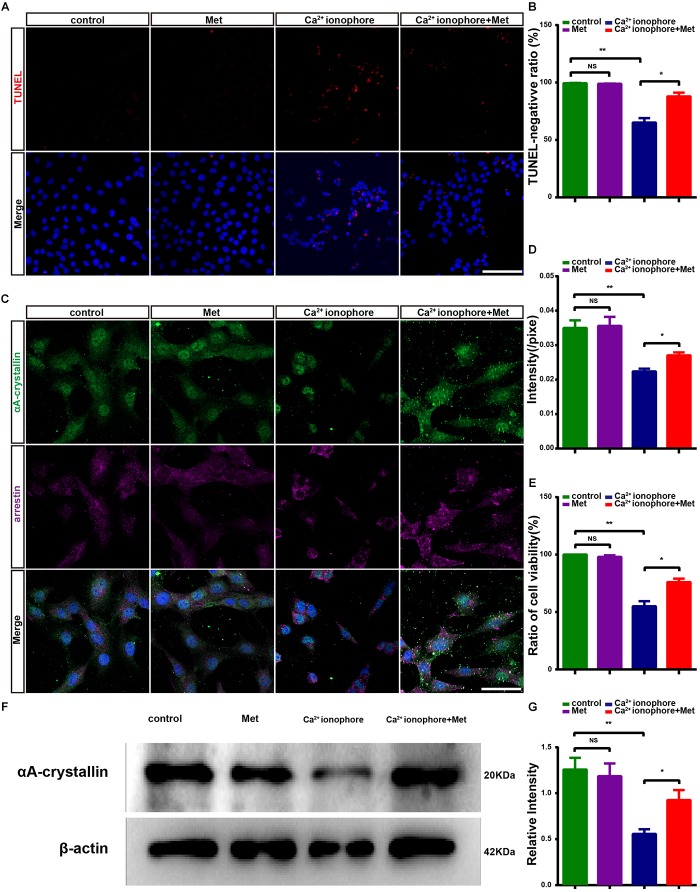
Metformin protects 661W cells from Ca^2+^ ionophore damage by increasing αA-crystallin expression. **(A)** Representative images of immunofluorescence for TUNEL (red) and DAPI (blue) in different groups: control; Met; Ca^2+^ ionophore; Ca^2+^ ionophore + Met. **(B)** Comparison of the number of TUNEL-negative cells in different groups. *n* = 5 samples per group. **(C)** Representative images of immunofluorescence for a 661W cell marker, anti-cone arrestin (purple) and αA-crystallin (green) and DAPI (blue) in different groups. **(D)** Comparison of the relative fluorescence intensities of αA-crystallin in different groups. *n* = 6 samples per group. **(E)** Comparison of cell viability in different groups in CCK8 assays. *n* = 5 samples per group. **(F)** Representative Western blot bands for αA-crystallin versus β-actin. **(G)** Comparison of protein grayscale semiquantitative analyses between the control group and the other three groups. *n* = 3 samples per group. Control: DMSO; Met: DMSO + metformin; Ca^2+^ ionophore: Ca^2+^ ionophore; Ca^2+^ ionophore + Met: Ca^2+^ ionophore + metformin. Data are presented as the mean ± SEM. NS, no significant difference. ^∗^*P* < 0.05, ^∗∗^*P* < 0.01. Scale bars: **(A)** 100 μm; **(D)** 50 μm.

It was previously reported that the Ca^2+^ ionophore (A23187, 2 lm, Sigma) induced Ca^2+^ overload in the 661W photoreceptor-like cell line and that Ca^2+^ overload induced the loss of phosphorylated CREB and calpastatin, thus activating calpain-2 and causing cell apoptosis, similar to the results that were observed in rd1 mice (Arroba et al., 2009). We treated 661W cells with Ca^2+^ ionophore to further verify the protective effect of metformin. The results showed that Ca^2+^ ionophore treatment significantly increased the number of suspended cells in the visual field and that the protruding area of the adherent cells was reduced in size and wrinkled (Supplementary Figure S4). In addition, the addition of metformin improved the survival of Ca^2+^ ionophore-treated 661W cells. These results demonstrate that the proportion of TUNEL-negative cells was not significantly different between the Met group and the control group (*P* > 0.05). However, the proportion of TUNEL-negative cells was significantly lower in the Ca^2+^ ionophore-treated group than in the control group (*P* < 0.01), and the increase in Ca^2+^ ionophore-induced cell apoptosis was significantly reduced by metformin (*P* < 0.05, *n* = 5) (Figure 8A,B). These results indicate that metformin protected the photoreceptor cells from the damage caused by Ca^2+^ overload.

To further demonstrate the protective effect exerted by metformin on photoreceptor cells, we examined the activity of 661W cells using CCK8 assays. There was no significant difference in cell viability between the control group and the Met group (*P* > 0.05). However, the cell viability in the Ca^2+^ ionophore-treated group was significantly lower than that in the control group (*P* < 0.01), and metformin treatment significantly decreased this damage (*P* < 0.05, *n* = 5) (Figure 8E). These results suggest that metformin protected photoreceptor cells from calcium overload-induced apoptosis *in vitro*.

### Metformin Upregulates the Expression of αA-Crystallin in 661W Cells

*In vitro*, metformin has a protective effect against Ca^2+^ ionophore-induced damage in 661W cells. To assess what happens to the crystallin in this process, we studied the changes in the expression of αA-crystallin in the control, Met, Ca^2+^ ionophore and Ca^2+^ ionophore + Met groups. The cell marker arrestin was used to specifically label 661W cells, and the results showed that in 661W cells, αA-crystallin was expressed and distributed throughout the cells (Figure 8C) (Hamann et al., 2013). The results also demonstrated that there was no significant difference in the average αA-crystallin fluorescence intensity between the Met group and the control group (*P* > 0.05) while it was significantly lower in the Ca^2+^ ionophore-treated group than in the control group (*P* < 0.01). However, the αA-crystallin levels in Ca^2+^ ionophore and metformin co-treated cells were significantly higher than those in Ca^2+^ ionophore-treated 661W cells (*P* < 0.05, *n* = 6) (Figure 8D) (Rao et al., 2012; Murakami et al., 2013). The Western blotting results were consistent with the immunofluorescence results (*P* < 0.01; *P* < 0.05, *n* = 3) (Figure 8F,G). Therefore, metformin may protect the photoreceptor-like cells, 661W, from Ca^2+^ ionophore-induced damage by increasing the expression of crystallin proteins.

## Discussion

Metformin is a widely used antidiabetic drug that has been observed to produce anti-inflammatory, antiapoptotic, and neuroprotective effects in a variety of neurodegenerative diseases, such as Alzheimer’s disease and Parkinson’s disease. In the present study, we show that intravitreally injected metformin produced an obvious protective effect on the visual function of rd1 mice at P14 and P18 and that this protective effect gradually decreased at P22. This finding suggests that metformin can delay but not completely prevent the progression of RD in rd1 mice. The effect of metformin is mainly mediated through the reduction in apoptosis of photoreceptor cells, inhibition of microglial activation and promotion of anti-inflammatory factors in the retina. Interestingly, metformin also increased the level of crystallin proteins, which are widely considered to exert anti-inflammatory and neuroprotective effects on the degenerated retina (Safe et al., 2018).

Metformin has been reported to produce antiapoptotic effects in several neurodegenerative diseases. For example, metformin was observed to block cellular apoptosis induced by tert-butyl hydroperoxide in nucleus pulposus cells, partially by activating autophagy (Chen D. et al., 2016; Hussein et al., 2018). In the present study, we observed that metformin produced an antiapoptotic effect on photoreceptors in degenerated retinas, an effect that was consistent with previous reports exploring the retina in diabetic mice, in which metformin was demonstrated to decrease retinal cell death in streptozotocin-induced diabetic mice by suppressing O-linked b-*N*-acetylglucosamine transferase (OGT) levels (Kim et al., 2017). However, unlike what is observed in diabetic retinopathy, RP is caused by inherited mutations in more than 95 genes that result in the loss of protein function, thus causing apoptosis in photoreceptors or RPE cells. As an AMPK activator, metformin has been demonstrated to improve the synthesis and folding of P23H rhodopsin *in vitro*; however, metformin also appeared to impair photoreceptor function, thus causing cell death in photoreceptors. This effect might result from the instability of metformin-rescued P23H rhodopsin (Athanasiou et al., 2017; Kim et al., 2017).

In contrast with the P23H RP mouse model, rd1 mice carry mutations in the β subunit of rod cGMP-phosphodiesterase (PDE6β); these mutations result in the accumulation of cGMP in the retina (Bowes et al., 1990; Wang et al., 2017). This accumulation affects the cyclic nucleotide-gated (CNG) channels in the outer segment membrane and causes deleterious calcium (Ca^2+^) influx, and the calcium overloading causes rapid rod cell death (Paquet-Durand et al., 2011). On the other hand, the accumulation of cGMP also activates cGMP-dependent protein kinase (PKG), which plays a key role in photoreceptor degeneration and early-onset severe retinal degeneration in rd1 mice (Paquet-Durand et al., 2009). In the present study, metformin produced antiapoptotic effects in photoreceptors and thereby delayed functional impairment in rd1 mice. Interestingly, our RNA-Seq analysis results indicated that crystallin proteins, which belong to the small heat shock protein (HSP) superfamily, are the target genes that are upregulated by metformin. Crystallin proteins, including αA-crystallin, exert characteristic antiapoptotic and cytoprotective effects against RD and its associated damage, especially by enhancing the survival of retinal ganglion cells (RGCs) in glaucoma or optic nerve crush (Piri et al., 2016). Our results show that 16 of the top 17 genes that showed the most significant differences in gene expression between Met and control mice were different subtypes of crystallin proteins. Furthermore, we found that αA-crystallin was expressed at significantly higher levels in the retina in the Met group than in the PBS-treated group. Therefore, to further confirm the effect of metformin, we also performed *in vitro* experiments in which 661W cells, once considered a mouse photoreceptor-like cell line but now considered a retinal ganglion precursor-like cell line (Sayyad et al., 2017), were treated with the Ca^2+^ ionophore. The results showed that metformin markedly increased the level of αA-crystallin and rescued the 661W cells from apoptosis. 661W cell lines used to be regarded as a cell model of cone cells and it has been shown that Ca^2+^ ionophore treatment caused the cell apoptosis which is similar to photoreceptor degeneration (Arroba et al., 2009), however, recent results demonstrated that 661W is more a RGCs-like cell than photoreceptor-like cell despite it expresses several cone markers (Sayyad et al., 2017). As the culture of primarily isolated photoreceptors is still great challenge, it is urgent to establish a new cell line of photoreceptors.

Xu et al. (2018) recently reported that subcutaneously injected metformin protects photoreceptors in the rd10 mice model by increasing mitochondrial biogenesis and suppressing oxidative stress through the AMPKα2 pathway in the neural retina. As metformin can cross the blood–retina barrier, daily subcutaneous injections demonstrated the ability to prolong rod survival in rd10 mice. However, Athanasiou et al. (2017) reported that metformin increased photoreceptor cell death in the P23H RP mouse model. The differences might have resulted from the different pathological mechanisms between the rd10 and P23H mouse models.

The present results showed that the amplitude of a-wave and b-wave at strong light is significantly improved by Metformin in rd1 mice at P14 and P18, while the latency did not improve significantly. However, the amplitude of a- and b-wave in metformin-treated group was still lower than that in C57 mice. It indicated that metformin only partly restored the retinal function by delaying but not stopping the degeneration of some photoreceptors in rd1mice. The degeneration of photoreceptors in rd1 is caused by the mutation of PDE6β and it develops quickly (Bowes et al., 1990), intravitreally injection of Metformin at P6, P9, and P12 partially rescued the photoreceptors in rd1 mice, and the neuroprotection of Metformin gradually vanished at P22 as it was cleared. Other cells such as bipolar cells in the degenerative retina seemed not to be protected by intravitreally injected Metformin, thus the latency of a-wave and b-wave was not improved in present study. Long-term metformin treatment might be taken into consideration in the treatment of retinal degeneration diseases (Deng et al., 2016). Our results also demonstrate that intravitreally injected metformin significantly upregulates the expression of crystallin, anti-inflammatory factors and antiapoptotic genes in the retina of rd1 mice. These proteins play a role in delaying RP to protect photoreceptors. Based on the literature regarding the effects of elevated levels of crystallin proteins, which include neuroprotection and the inhibition of inflammation and apoptosis, we speculate that metformin may play a role in promoting the expression of crystallin proteins to inhibit inflammation and apoptosis. However, whether metformin directly affects anti-inflammatory and antiapoptotic effects or exerts anti-inflammatory and antiapoptotic effects indirectly through elevated crystallin protein levels and the mechanism by which metformin upregulates the expression of crystallin proteins in RP are issues that need exploration in future studies.

Our study demonstrates that intravitreally injected metformin can delay the progression of RP in rd1 mice. In addition, we provide the first data suggesting that the functional protective effects of metformin may be through neuroprotection and the inhibition of inflammation by increasing the expression of crystallin proteins. Metformin and crystallin proteins may therefore be used in the treatment of RP in the future. Our study provides new directions and ideas for future research on RP, and we have identified new possibilities for the use of metformin in retinal diseases.

## Ethics Statement

The study was approved by the Institutional Animal Care and Use Committee of Third Military Medical University, Chongqing, China.

## Author Contributions

LA: conception and design, collection and assembly of data, data analysis and interpretation, and manuscript writing. TZ, JH, XC, and DS: collection and assembly of data and provided assistance during the experiment to ensure the objectivity of the experimental process. XF: conception and design, manuscript writing, and final approval of the manuscript. HX: conception and design, manuscript writing, financial support, data analysis and interpretation, and final approval of the manuscript.

## Conflict of Interest Statement

The authors declare that the research was conducted in the absence of any commercial or financial relationships that could be construed as a potential conflict of interest.

## Footnotes

1 https://sph.uth.edu/retnet

2 https://www.kegg.jp

3 http://www.genome.jp/kegg/

4 http://www.geneontology.org/
