# Supplementary material for: Rescue of Retinal Degeneration in rd1 Mice by Intravitreally Injected Metformin
Source: Front Mol Neurosci. 2019 Apr 26;12:102. doi: 10.3389/fnmol.2019.00102 (PMC6497809; doi:10.3389/fnmol.2019.00102)
Supplement: Supplementary file 1 [file Image_1.pdf]

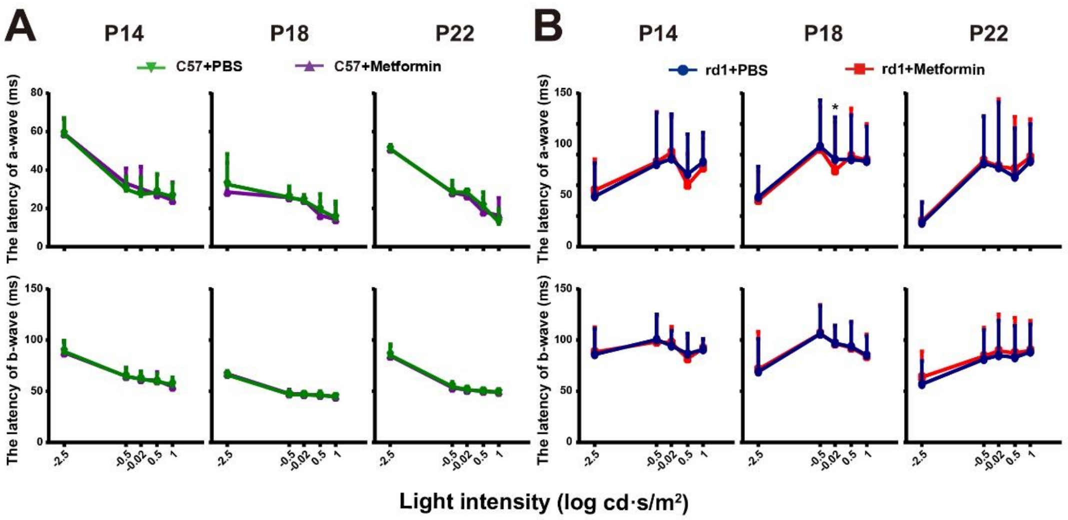

Supplementary material Figure **1**: Metformin has little effect on the latency of a- and b-wave in C57 and rd1 mice. Comparison the latency of a- and b-wave in the control and metformin treatment groups at different periods. **A**. C57 mice; **B**. rd1 mice. *P < 0.05. n = 5 mice per group.

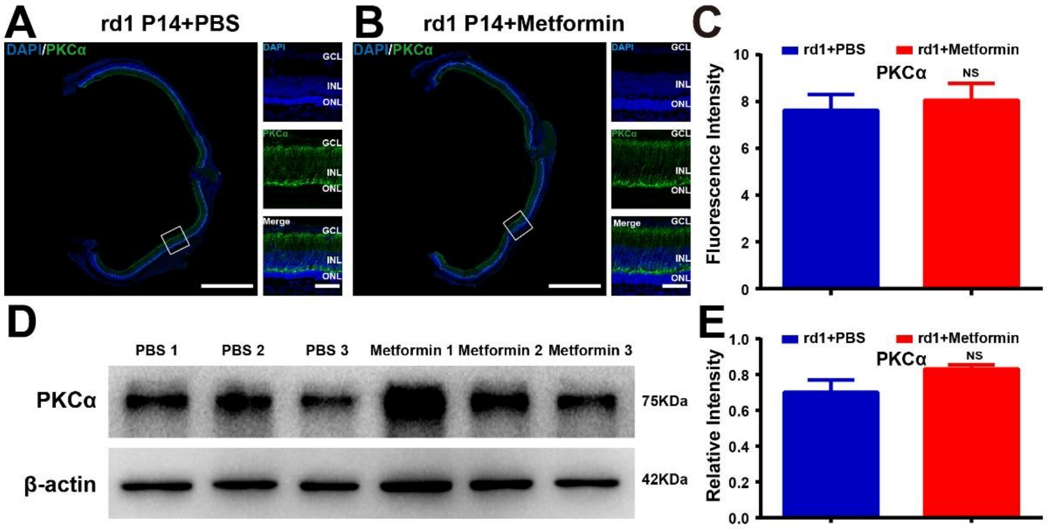

Supplementary material Figure **2**. metformin has no significant effect on bipolar cells in the retina of rd1 mice at P14. **A, B.** Low magnification field of view and enlarged view of PKCα in the middle of the retina in the control group (A) and the Met group (B). **C.** Comparison of the relative fluorescence intensities of PKCα in approximately 400 μm from the optic papilla. n = 5 mice per group. **D.** Representative Western blot bands of PKCα versus β-actin. **E.** Comparison of a protein grayscale semiquantitative analysis between the control group and the Met group. n = 3 mice per group. GCL, retinal ganglion cell layer; INL, inner nuclear layer; ONL, outer nuclear layer. Data are presented as the mean ± SEM. NS, no statistical difference. Scale bar: whole retinal map, 500 μm; enlarged view 50 μm.

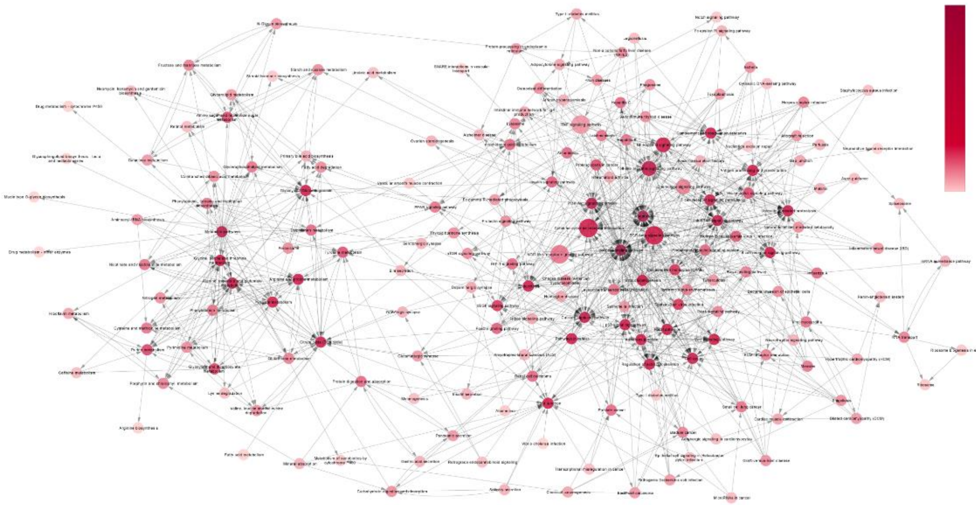

Supplementary material Figure **3**: Pathway-act-network analysis of the retina in metformin-treated and PBS-treated rd1 mice at P14. A pathway-act network was constructed according to the interactions within pathways identified in the KEGG database. Each node (red circle) represents a signaling pathway. Arrows represent interactive relationships between two signaling pathways.

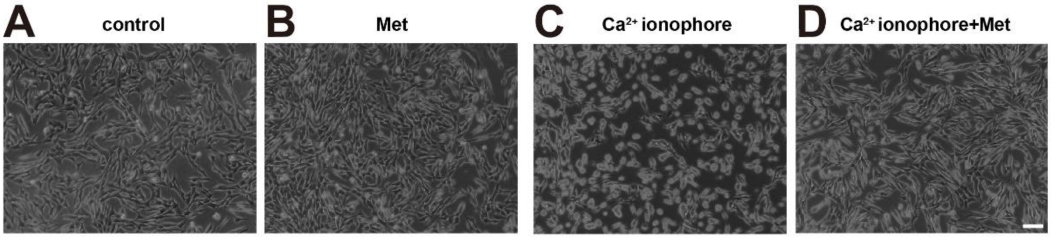

Supplementary material Figure **4**: Representative images of different treatments of 661W in the visual field. Scale bars: 100 μm.
